# Impact of Supine, Standing, and Sitting Postures on Acetabular Component Orientation in Total Hip Arthroplasty

**DOI:** 10.7759/cureus.77279

**Published:** 2025-01-11

**Authors:** Vignesh Veluswamy, Rajarajan Nagu Sakthivel

**Affiliations:** 1 Department of Orthopaedics, Employees' State Insurance Corporation (ESIC) Medical College and Hospital, Chennai, IND

**Keywords:** acetabulum fracture fixation, craniocervical alignment, developmental dysplasia of the hip, osteoarthritis, total hip arthroplasty (tha)

## Abstract

Introduction: Total hip arthroplasty (THA) is a highly effective surgical procedure aimed at relieving pain and restoring function in patients with severe hip joint disorders, such as osteoarthritis, osteonecrosis, and developmental dysplasia of the hip (DDH). The primary goals of THA are to alleviate pain, improve joint function, and enhance the quality of life by providing a stable and well-functioning hip joint. Accurate orientation of the acetabular component is critical for the success of THA, ensuring optimal joint mechanics, reducing the risk of complications such as dislocation and wear, and enhancing implant longevity. Postural changes significantly influence the orientation and stability of the acetabular component. Traditional supine evaluations may not accurately reflect the functional alignment during daily activities, necessitating more dynamic assessment methods.

Materials and methods: The study was conducted at a tertiary care hospital in Chennai, Tamil Nadu, India, from March 2018 to October 2019. It was a prospective study involving 27 patients who underwent primary THA. Exclusion criteria included bilateral or revision THA, a history of spine or hip surgery, neurological or musculoskeletal disorders, or lower limb deformities. Computed tomography scans were performed in supine, simulated standing, and sitting postures. Pre-operative and post-operative evaluations included standing and sitting lateral lumbosacral spine radiographs. Measurements at the spinopelvic junction evaluated spinal balance and pelvic compensation. The anatomical acetabular anteversion (AAA) was determined in supine, standing, and sitting positions using CT scans.

Results: The study included 27 patients aged 19 to 66 (mean age 37). Most patients (17, 63%) were between 21-40 years old. Osteoarthritis was the most common indication for THA (15, 55.6%), followed by osteonecrosis (six, 22.2%), DDH (four, 14.8%), and post-traumatic arthritis (two, 7.4%). Spinopelvic mobility assessment showed 15 (55.6%) participants with normal mobility, eight (29.6%) who were hypermobile, three (11.1%) stiff, and one (3.7%) who were severely stiff. Significant variability in acetabular component orientation was observed across postures: mean supine AAA was 11.04° (SD 14.44°), standing AAA was 13.07° (SD 14.38°), and sitting AAA was 30.93° (SD 14.28°). The mean difference between sitting and standing AAA was 17.85° (SD 11.83°). Statistical analysis revealed significant differences in acetabular component orientation across different postures (p < 0.05).

Conclusion: This study highlights the critical need for dynamic and individualised approaches in THA. The findings underscore the importance of dynamic pre-operative assessments, including standing and sitting radiographs, to optimise component placement. Clinical recommendations include tailoring surgical techniques based on individual spinopelvic mobility, utilising advanced intraoperative tools for precise placement, and developing customised post-operative rehabilitation programs.

## Introduction

Total hip arthroplasty (THA) is a successful surgical treatment for people with severe hip joint problems, including developmental dysplasia of the hip (DDH), osteoarthritis, and osteonecrosis. It also relieves pain and restores function. During the procedure, a prosthetic implant, usually consisting of femoral and acetabular components, is used to replace the injured hip joint. By providing patients with a secure and functional hip joint, THA aims to reduce pain, improve joint function, and improve the patient's quality of life [1].

Accurate alignment of the acetabular component is not only necessary but essential to the success of THA. This part's precise positioning guarantees ideal joint mechanics, lowers the possibility of wear, impingement, and dislocation, and lengthens the implant's lifespan. On the other hand, improper placement increases the risk of implant failure, accelerates the wear of the prosthetic components, and results in an unequal distribution of load. The possible dangers of incorrect acetabular component orientation are highlighted by our research, underscoring the necessity of exact surgical methods and careful pre-operative preparation [2]. The acetabular component's stability and alignment are greatly impacted by variations in posture. The majority of traditional component positioning assessments are carried out while the patient is in a supine position. Nonetheless, standing and sitting are common daily actions that can change the hip joint's position and the way the femoral and acetabular components are related [3].

In the supine position, the hip's functional alignment during activities like walking or sitting is not effectively represented by the supine position, which is frequently utilised for radiography examinations. This disparity may result in an implant location that is not ideal and does not take dynamic variations in hip joint mechanics into consideration. Our research elucidates the limitations of the supine position and advocates for more precise assessment techniques that take into account the patient's real postures during activities of daily living. In a standing position, the location of the acetabular component can be affected by changes in the orientation of the pelvis and spine during standing. To make sure the implant functions properly when bearing weight, the body's alignment in this posture and the forces of gravity must be taken into account.

Sitting position frequently causes the hip joint to flex significantly, which modifies the spatial arrangement of the femoral and acetabular components. Because many dislocations happen when patients go from sitting to standing or the other way around, this posture is especially important. Such issues can be anticipated and avoided with the use of acetabular orientation evaluation in the sitting position. Assessing the orientation of the acetabular component in these diverse positions is crucial for comprehending the implant's functionality under varied circumstances. A more thorough examination can be obtained through dynamic assessment with imaging techniques such as computed tomography (CT) in simulated standing and sitting postures. This can improve surgical outcomes and lower the risk of postoperative problems [4]. The primary objective of the study is to evaluate how different postures affect acetabular component orientation.

## Materials and methods

The study was conducted at Sri Ramachandra Institute of Higher Education and Research, Chennai, India, from March 2018 to October 2019, after receiving ethical committee clearance from Sri Ramachandra Medical College and Research Institute's Ethical Committee (approval number: MED/18/FEB/42/43), a prospective study including 27 patients who underwent primary THA. Patients with bilateral or revision THA, a history of spine or hip surgery, neurological or musculoskeletal disorders, or lower limb deformities were excluded from the study. Computed tomography scans were performed for all the patients in supine, simulated standing, and sitting postures. Pre-operative and post-operative evaluations included standing and sitting lateral lumbosacral spine radiographs to assess the spine-pelvis-hip relationship.

Patients were evaluated with standing and sitting lateral lumbosacral spine radiographs that included the pelvis. The spine-pelvis-hip relationship was captured on a lateral radiograph that included the L3 vertebra to the proximal part of the femur. The lower three lumbar vertebrae significantly influence the hip; thus, the radiographs were taken in a standing position where the hip was positioned next to the cassette in a relaxed posture, with the patient's arms folded across the chest resting at a 90° angle on support to minimise trunk posture variation. The X-ray beam was centred over the L4-L5 junction, perpendicular to the patient's axial line, and the source-to-film distance was 183 cm. In a sitting position, the patient was made to sit on a stool with a straight back, no trunk pressure against the back of the chair, and a relaxed posture. The angle between the thighs and trunk was 110° to allow better visualisation of the pubic symphysis and provide a comfortable sitting position. Measurements at the spinopelvic junction evaluated spinal balance and pelvic compensation.

Pre-operative lateral spinopelvic radiographs provided skeletal measurements of pelvic incidence and sacral slope using Synapse PACS (Fujifilm Healthcare Americas Corporation, Lexington, MA, USA). Immediate post-operative radiographs were taken, followed by conventional CT scans at three to six weeks post-operatively. Acetabular component orientation was measured using CT scans. The anatomical acetabular anteversion (AAA) was determined in supine, standing, and sitting positions by adjusting the sectional plane to the sacral slope. Inclination and anteversion were calculated using established radiographic techniques.

## Results

**Figure 1:**
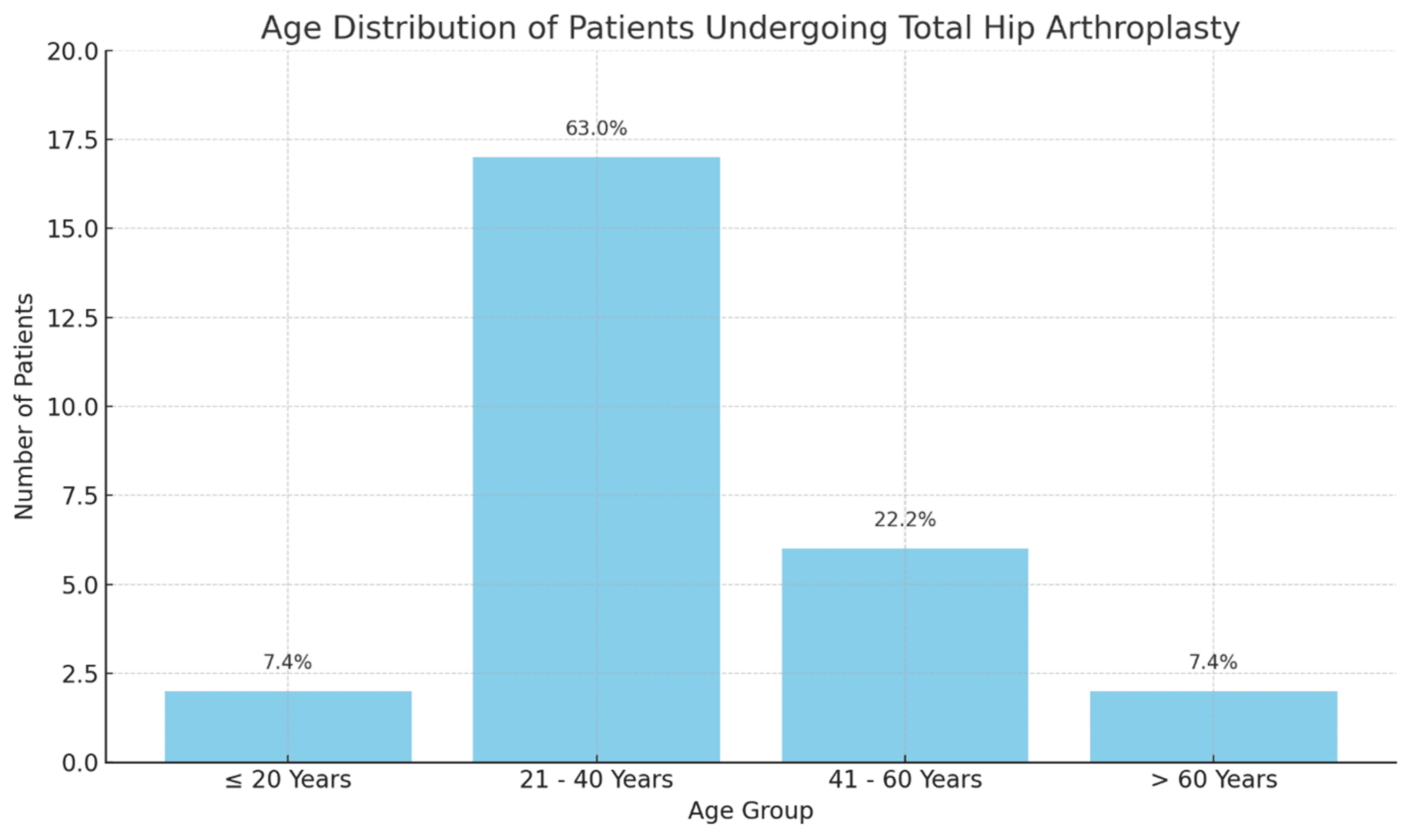
Age-wise distribution of patients who underwent total hip arthroplasty (THA)

The study included 27 patients who underwent primary THA. The age distribution of the patients ranged from 19 to 66 years, with a mean age of 37 years. Most patients (17, 63%) fell within the 21-40-year age group, indicating that this demographic is most commonly affected by the conditions necessitating THA in our study population. This age distribution suggests that younger adults are increasingly undergoing THA, potentially due to early-onset osteoarthritis or other hip disorders​​ as shown in Table 1 and Figure 1.

**Table 1:** Age distribution of the patients who underwent primary total hiparthroplasty (THA) The data have been represented as numbers (N) and percentages (%).

Age group	Number of patients (N)	Percentage (%)
≤ 20 years	2	7.4
21-40 years	17	63.0
41-60 years	6	22.2
> 60 years	2	7.4
Total	27	100.0

**Figure 2:**
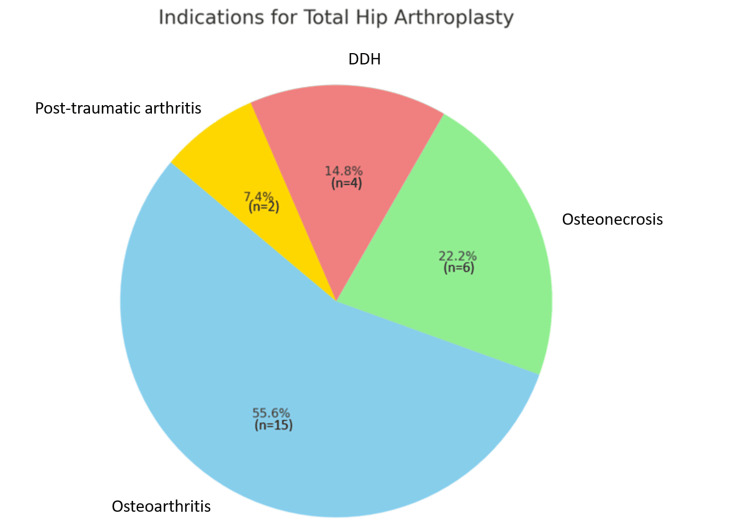
Indications for total hip arthroplasty (THA) DDH: developmental dysplasia of the hip

Osteoarthritis emerged as the most common indication, accounting for 55.6% (15 patients) of the cases. This highlights the significant impact of osteoarthritis on hip function and the frequent need for surgical intervention to alleviate pain and restore mobility. Osteonecrosis was the second most prevalent indication, representing 22.2% (six patients), emphasising the severity of this condition, which often leads to bone tissue death due to lack of blood supply and necessitates THA when conservative treatments fail. Developmental dysplasia of the hip, a congenital condition affecting hip joint formation, accounted for 14.8% (four patients) of the cases, reflecting the long-term degenerative changes and joint dysfunction resulting from this developmental disorder. Post-traumatic arthritis, arising from joint injury and subsequent cartilage damage, was the least common indication, comprising 7.4% (two patients) of the cases. This distribution of indications provides valuable insight into the primary reasons for THA, with osteoarthritis being the predominant cause as shown in Table 2 and Figure 2.

**Table 2:** Indications for total hip arthroplasty (THA) The data have been represented as numbers (N) and percentages (%).

Indication for THA	Number of patients (N)	Percentage (%)
Osteoarthritis	15	55.6
Osteonecrosis	6	22.2
Developmental dysplasia of the hip (DDH)	4	14.8
Post-traumatic arthritis	2	7.4
Total	27	100.0

**Figure 3:**
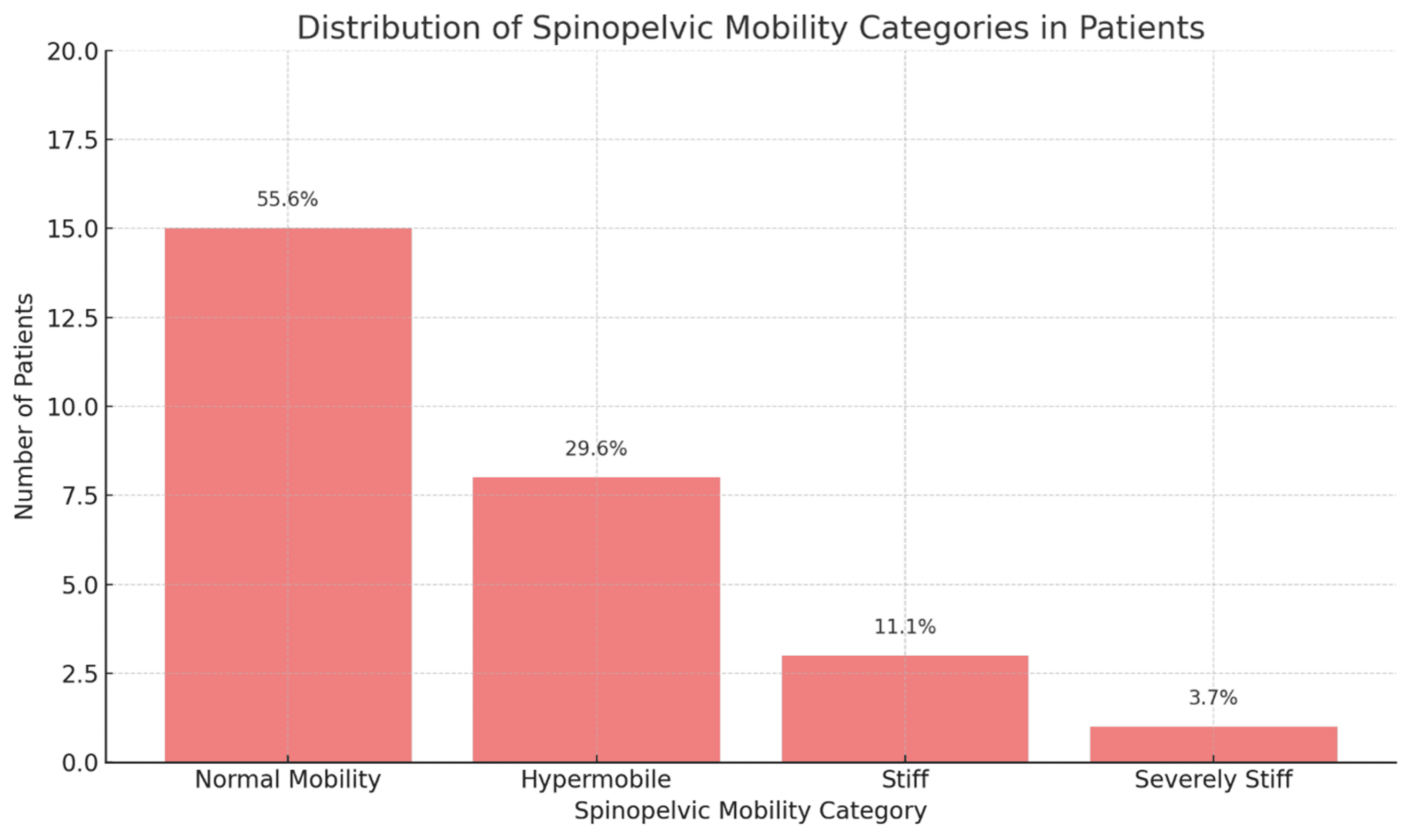
Spinopelvic mobility categories

Spinopelvic mobility in patients was assessed and categorised into four distinct groups based on radiographic evaluations. Normal mobility was observed in 15 patients (55.6%), who exhibited typical ranges of motion and alignment in the pelvic and lumbar regions. Eight patients (29.6%) were classified as hypermobile, indicating an above-average range of motion at the spinopelvic junction, which could potentially impact the stability of the acetabular component during postural changes. Three patients (11.1%) were noted to have stiffness, characterised by limited movement between standing and sitting postures, increasing the risk of impingement and dislocation. One patient (3.7%) was identified as severely stiff, showing minimal to no movement at the spinopelvic junction, which poses significant challenges in maintaining the stability and alignment of the acetabular component following THA as shown in Table 3 and Figure 3.

**Table 3:** Spinopelvic mobility categories The data have been represented as numbers (N) and percentages (%).

Spinopelvic mobility category	Number of patients (N)	Percentage (%)
Normal mobility	15	55.6
Hypermobile	8	29.6
Stiff	3	11.1
Severely stiff	1	3.7
Total	27	100.0

**Figure 4:**
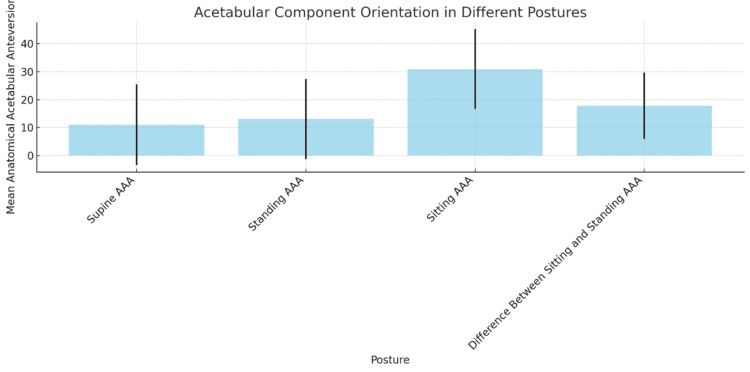
Acetabular component orientation AAA: anatomical acetabular anteversion

The orientation of the acetabular component was measured in three different postures: supine, sitting, and standing, and showing significant variability across these positions. In the supine position, the mean AAA was 11.04°, with a wide standard deviation of 14.44°, indicating considerable variation in how the acetabular component is aligned when patients are lying down, the standard pre-operative posture. In the standing position, the mean AAA increased slightly to 13.07° with a standard deviation of 14.38°, reflecting changes in pelvic tilt and orientation when the body is bearing weight. In the sitting posture, the AAA increased significantly to 30.93°, with a standard deviation of 14.28°, highlighting the effects of hip flexion and pelvic tilt on acetabular orientation, which is critical for assessing dislocation and impingement risks during activities like sitting or standing up. The mean difference in AAA between sitting and standing was 17.85° (standard deviation of 11.83°), emphasizing the importance of considering dynamic postural changes when planning for acetabular component placement as shown in Table 4 and Figure 4.

**Table 4:** Acetabular component orientation p-value < 0.05 is considered statistically significant. The data have been represented as Mean ± SD.

Posture	Mean (°)	Standard deviation (°)	N	t-value/F-value	p-value
Supine anatomical acetabular anteversion (AAA)	11.04	14.44	27	-	0.000
Standing AAA	13.07	14.38	27	-	0.000
Sitting AAA	30.93	14.28	27	-	0.000
Difference between sitting and standing AAA	17.85	11.83	27	t = 3.21	0.001

Statistical analysis confirmed significant differences in orientation across these postures (p < 0.05), with the cup being 17.86° more anteverted on average when transitioning from standing to sitting, underscoring the need for multi-postural assessments to enhance pre-operative planning and post-operative outcomes as shown in Table 5. 

**Table 5:** P-values for the comparisons of acetabular component orientation across different postures p-value < 0.05 is considered statistically significant.

Comparison	t-value/F-value	p-value
Supine vs. standing	t = 2.14	0.000
Standing vs. sitting	t = 3.21	0.000
Supine vs. sitting	t = 4.00	0.000

The study highlighted significant dynamic pelvic motion, with the sacral tilt being approximately 40° in the standing position and reducing to 20° in the sitting position, showing a 20° difference between these postures. This dynamic change correlates with the femur's extension relative to the pelvis when standing and its flexion when sitting. It was also observed that unilateral THA does not notably affect spinal alignment, preserving the natural dynamic motion of the spinopelvic junction. Both pre-operative and post-operative assessments showed minimal changes in sacral slope, confirming that pelvic alignment remains consistent before and after surgery.

Pelvic incidence is defined as a static parameter representing the spine’s axis of sagittal balance. It is measured as the angle between a line perpendicular to the superior endplate of S1 at its midpoint and a line connecting that point to the femoral head axis, with normal values ranging from 42° to 63° and an average of 50° ± 10°. Sacral tilt, also referred to as sacral slope, normally measures 40° when standing and reduces to 20° when sitting, demonstrating the pelvis’s dynamic motion. Degenerative disc disease was identified in patients using a grading system assessing facet arthrosis, disc height narrowing, and endplate changes, with the condition being confirmed by the presence of two or more affected discs as shown in Table 6.

**Table 6:** Pre-operative and post-operative radiographic findings

Parameter	Descripton	Values
Pelvic motion	Dynamic motion of the pelvis, with sacral tilt changing between standing and sitting.	40° (standing) → 20° (sitting)
FemurpPosition	Relative to the pelvis, the femur is extended when standing and flexes when sitting.	Changes based on posture
Spinal alignment (total hip arthroplasty (THA))	Unilateral THA does not significantly alter spinal alignment, preserving the natural dynamic motion of the spinopelvic junction.	Consistent pre- and post-surgery
Sacral slope	Angle between the superior end plate of S1 and a horizontal line, representing pelvic orientation. No significant change pre- and post THA.	40° (standing) → 20° (sitting)
Pelvic incidence	A static parameter defining the sagittal balance of the spine, measured from the S1 midpoint to the femoral head axis.	42° to 63° (normal range), 50° ± 10°
Sacral tilt	Another term for sacral slope, indicating the dynamic pelvic motion between standing and sitting positions.	40° (standing) → 20° (sitting)
Degenerative disc disease	Defined as the presence of two or more diseased discs, assessed through facet arthrosis, disc height narrowing, and endplate proliferative changes.	Based on specific degenerative changes

## Discussion

The study's findings shed important light on spinopelvic mobility, acetabular component orientation, patient demographics, and indications for THA. The age distribution of THA patients in this research suggests that hip diseases requiring surgery are increasingly affecting younger persons, especially those between the ages of 21 and 40. This age group's prevalence (63%) points to a possible shift in the development of illnesses like osteoarthritis and osteonecrosis, which may be brought on by genetic predispositions or lifestyle choices. The need for specialised surgical methods and long-term follow-up plans to address the particular difficulties younger THA patients encounter is highlighted by this demographic shift.

As in prior research, osteoarthritis accounted for 55.6% of THA cases in this study, making it the most common indication. This is consistent with research by Kurtz et al., which found that osteoarthritis accounts for about 58% of THA cases in the US and is the primary cause of the condition [5]. Osteonecrosis, on the other hand, was the second most frequent sign, indicating the serious damage this illness causes to the integrity of the hip joint. Osteonecrosis is responsible for roughly 10%-12% of THA surgeries, according to Mont et al. [6], a somewhat lower rate than what was found in this study.

With 55.6% of patients, osteoarthritis was the most common reason for THA. This discovery is consistent with previous research, which emphasises osteoarthritis as a primary contributor to hip discomfort and dysfunction. Osteonecrosis is represented by a large percentage (22.2%), highlighting the serious impact this illness has on hip joint integrity and the need for surgical intervention when conservative treatments fail. Though less common, post-traumatic arthritis and DDH both demonstrate the variety of underlying disorders that can cause THA. The diverse range of indications highlights the significance of customised pre-operative planning and patient education in order to maximise results for various patient populations.

Consistent with the literature, post-traumatic arthritis and DDH were less common indications. Due to the early development of degenerative alterations, Hartofilakidis et al. pointed out that THA is frequently performed on DDH patients at a younger age [7]. The results of this study, which showed that 63% of patients were between the ages of 21 and 40, are consistent with the patterns noted by Glyn-Jones et al. [8], who noted that a growing proportion of younger adults were receiving THA because of ailments such as DDH and early-onset osteoarthritis.

According to the spinopelvic mobility assessment, a significant percentage of patients (29.6%) were classed as hypermobile, whilst the majority of patients (55.6%) showed normal mobility. The stability of the acetabular component may be impacted by hypermobility at the spinopelvic junction, especially during postural alterations. Additional problems for THA arise from patients with stiffness (11.1%) or severe stiffness (3.7%), as limited movement of the spine might increase the risk of impingement and dislocation. These results emphasise the necessity of comprehensive pre-operative assessments in order to guide component location and evaluate spinopelvic dynamics.

The study included 27 patients (N=27), with 55.6% (N=15) exhibiting normal spinopelvic mobility, 29.6% (N=8) classified as hypermobile, 11.1% (N=3) stiff, and 3.7% (N=1) severely stiff, as determined by the measurement of spinopelvic mobility in this study, was found. These results are consistent with those published by Lazennec et al. [1], who highlighted the significance of assessing spinopelvic dynamics in order to avoid problems like impingement and dislocation. The study's findings, which indicate that hypermobility at the spinopelvic junction can have a major impact on THA outcomes, are corroborated by the prevalence of hypermobility seen in the research conducted by Esposito et al. [9].

The study showed that the direction of the acetabular component varied significantly between positions. A broad standard deviation of 11.04° for the mean supine AAA suggests variability in posture during routine pre-operative evaluations. The impact of pelvic tilt and hip flexion on component orientation is shown by the rise in mean AAA to 13.07° in the standing position and a significant 30.93° in the sitting position. The 17.85° mean difference between standing and sitting postures highlights how crucial it is to take dynamic variations in posture into account for precise component placement. The results of the statistical analysis demonstrated that there were substantial orientation differences between postures (p < 0.05), indicating that dynamic radiography evaluations are essential for pre-operative planning.

The substantial variation in acetabular component orientation between postures that this study found is consistent with other studies' findings. Similar results were noted by Lazennec et al. [4], who pointed out that the anteversion is frequently underestimated in the supine position as opposed to the standing and seated positions. The ranges reported by Bhaskar et al. [10] are consistent with the mean supine AAA of 11.04°, which increases to 13.07° in the standing position and 30.93° in the sitting position. Their findings highlight the importance of dynamic evaluations for optimal component placement.

The significant 17.85° mean difference between sitting and standing positions emphasises how important hip flexion and pelvic tilt are for component orientation. This result is consistent with the findings of Dorr et al. [11], who showed the clinical implications of acetabular anteversion variability for THA stability. These disparities' statistical significance (p < 0.05) supports Redmond et al.'s recommendations for thorough pre-operative evaluations that incorporate dynamic radiography examinations [12].

Pre- and post-operative radiographic examinations revealed a consistent pelvic alignment, and measures of the sacral slope revealed a large amount of dynamic pelvic motion. The sacral tilt, which is typically 40° when standing and 20° when sitting, is a dynamic range that is necessary to preserve pelvic and spinal balance. Pre- and post-operative pelvic alignment is unaltered, indicating that unilateral THA preserves natural spinopelvic motion without appreciably changing spinal alignment. Furthermore, consistent pelvic alignment was indicated by the fact that pelvic incidence, a static measure of sagittal balance, stayed within normal ranges.

The results of Grammatopoulos et al., who found only minor changes in spinal alignment following total hip arthroplasty [13], are comparable with the stability of sacral slope measures pre-and post-operatively, with sacral tilt ranging from 40° while standing to 20° while sitting. The unaltered pelvic incidence values corroborate the findings of Ishida et al. [14], who emphasised the retention of normal spinopelvic mobility following THA. The results of this investigation have a number of significant ramifications for both surgical technique and component placement in THA. The observed variations in patient demographics, spinopelvic mobility, and acetabular component orientation in various postures give rise to these implications.

The significance of dynamic pre-operative examinations is highlighted by the notable variations in acetabular component orientation found in supine, standing, and sitting positions. The functional location of the acetabular component may not be fully depicted by traditional static imaging, which is usually done supine. Including dynamic assessments with sitting and standing radiographs can aid surgeons in comprehending how hip flexion and pelvic tilt affect component orientation. In order to improve post-operative results and lower the risk of problems such as impingement and dislocation, precise pre-operative planning and optimal component placement are essential [1].

The requirement for individualised methods for component placement is highlighted by the variation in spinopelvic mobility among patients. Normal spinopelvic mobility patients may benefit from routine procedures. However, to address the particular issues associated with hypermobility or stiffness, tailored techniques are required for people affected. Greater focus on the stability of the acetabular component during postural shifts is necessary for patients who are hypermobile. For individuals who experience stiffness, it is critical to ensure a sufficient range of motion without running the risk of impingement or dislocation. When choosing the kind and location of implants, surgeons ought to take these things into account [10].

Precise component placement can be accomplished during surgery with the aid of instruments like robotic-assisted surgery and computer-assisted navigation, which offer real-time input on the orientation of the acetabular component in relation to the patient's anatomy and posture. Surgeons can use these technologies to help them make the necessary changes to account for individual differences in pelvic tilt and spinopelvic mobility. Furthermore, to further guarantee ideal alignment and orientation, intraoperative imaging can be used to confirm component location in various postures [4].

Rehabilitation and post-operative care procedures can benefit from an understanding of the dynamic nature of spinopelvic mobility and acetabular component orientation. Patients who have notable variations in component orientation between sitting and standing, for example, might benefit from specialised physical therapy regimens that emphasise slow, deliberate movements to stabilise the hip joint. Timely interventions and better long-term results can result from continuously monitoring these individuals for indications of instability or problems [9]. The education and training of orthopaedic surgeons may be affected by these findings. Future surgeons might get the skills necessary to manage the complications of THA by emphasising in surgical training programmes the significance of dynamic assessments and individualised approaches. The utilisation of cutting-edge imaging and navigational technologies as well as methods for identifying and adjusting for changes in spinopelvic mobility should be covered in training [11].

The results of this study highlight the complexity of THA in a range of patient demographics, underscoring the importance of thorough pre-operative evaluations that take age, underlying reasons, spinopelvic mobility, and dynamic postural alterations into account. Subsequent studies ought to concentrate on optimising surgical methods to suit the distinct anatomical and functional attributes of younger total hip arthroplasty patients as well as those with notable changes in spinopelvic mobility. To assess the influence of these parameters on implant lifetime and patient outcomes, longer-term studies are also required.

This study has some limitations that should be acknowledged. The study has a small sample size of 27 patients, which limits the generalisability of the findings, and a larger cohort would provide more robust statistical power. Additionally, the short follow-up period of three to six weeks is insufficient to assess long-term outcomes such as implant wear, dislocation, or functional improvements, making it necessary to evaluate these factors over a longer term. The study also lacks functional outcome measures, such as patient-reported outcomes or physical function assessments, which limits the ability to correlate radiographic findings with clinical effectiveness. Furthermore, while the use of CT scans in simulated standing and sitting postures provides valuable anatomical data, these conditions may not fully reflect real-world postures during daily activities. The exclusion of patients with bilateral or revision THA, spine or hip surgery, and neurological or musculoskeletal disorders narrows the applicability of the results to more complex patient populations. As a single-centre study, the findings may not be generalisable to other institutions with different patient demographics or surgical techniques. Lastly, while spinopelvic mobility was categorised using radiographic assessments, these may not fully capture the dynamic nature of pelvic motion during functional activities, suggesting that future research should incorporate more dynamic imaging techniques and longer-term follow-up to strengthen the conclusions.

The present study emphasizes the crucial function of tailored pre-operative planning and dynamic radiographic evaluations in maximizing the results of THA. Surgeons can improve the stability, functionality, and durability of hip implants by tailoring their care to the unique requirements of various patient groups and comprehending the influence of spinopelvic dynamics.

## Conclusions

This study shows how important it is to use personalised, dynamic techniques while performing THA. Important discoveries show that younger folks make up the majority of patients and that osteoarthritis is the primary reason for surgery. Considerable variation in spinopelvic mobility and acetabular component orientation between postures highlights the need for dynamic pre-operative evaluations, which involve standing and sitting radiographs, in order to maximise component positioning. Clinical recommendations include designing individualised post-operative rehabilitation programs, utilising improved intraoperative instruments for precise placement, and customising surgical procedures depending on each patient's unique spinopelvic mobility. Subsequent investigations ought to concentrate on extended duration results, sophisticated imaging methods, spinopelvic mobility variability impacts, and comparisons between static and dynamic evaluations. Surgeons can decrease the risk of complications in THA, improve patient outcomes, and increase surgical precision by implementing these measures.
